# Platelet-Derived Growth Factor Induces *Rad* Expression through Egr-1 in Vascular Smooth Muscle Cells

**DOI:** 10.1371/journal.pone.0019408

**Published:** 2011-04-29

**Authors:** Yan Luo, Meiling Zhang, Ji Zhang, Jifeng Zhang, Chunlei Chen, Y. Eugene Chen, Jing-Wei Xiong, Xiaojun Zhu

**Affiliations:** 1 The Institute of Molecular Medicine, Peking University, Beijing, China; 2 The Cardiovascular Center, University of Michigan, Ann Arbor, Michigan, United States of America; Istituto Dermopatico dell'Immacolata, Italy

## Abstract

**Background:**

Ras associated with diabetes (Rad) inhibits vascular lesion formation by reducing the attachment and migration of vascular smooth muscle cells (VSMCs). However, the transcriptional regulation of Rad in VSMCs is unclear.

**Methodology and Principal Findings:**

We found that Platelet-Derived Growth Factor (PDGF)induced Rad expression in a time- and dose-dependent manner in rat aortic smooth muscle cells (RASMCs) using quantitative real-time PCR. By serial deletion analysis of the Rad promoter, we identified that two GC-rich early growth response-1 (Egr-1) binding sites are essential for PDGF-induced Rad promoter activation. Overexpression of Egr-1 in RASMCs strongly stimulated Rad expression while the Egr-1 corepressor, NGFI-A binding protein 2 (NAB2), repressed PDGF-induced Rad up-regulation in a dose-dependent manner. Direct binding of Egr-1 to the Rad promoter region was further confirmed by chromatin immunoprecipitation assays.

**Conclusions:**

Our results demonstrate that Rad is regulated by PDGF through the transcriptional factor Egr-1 in RASMCs.

## Introduction

Ras associated with diabetes (Rad) is a member of the RGK family which is composed of Rad, Gem/Kir, Rem and Rem2 [1]. It is expressed in the heart, skeletal muscle and lung [2]. Rad is highly expressed in the skeletal muscle of some type II diabetic patients [2], which suggests that Rad is related with glucose metabolism and insulin resistance. Our previous studies demonstrate that Rad is critical in maintaining normal cardiac functions. Rad expression decreases significantly in human failing hearts and Rad knockout (KO) mice are more susceptible to cardiac hypertrophy with increased CaMKII phosphorylation compared with their littermate controls [3]. Our findings as well as the others' indicate that Rad inhibits myocardium L-type calcium channel activity and attenuates the β-Adrenergic Receptor (β-AR) activity [4], [5]. Dominant negative suppression of endogenous Rad in the heart up-regulate L-type Ca^2+^ channel expression on the plasma membrane, leading to I_Ca,L_ increase and action potential prolongation [6]. Rad is upregulated in vascular smooth muscle cells (VSMCs) during the formation of vascular lesions and overexpression of Rad attenuated neointimal formation by strongly inhibiting smooth muscle migration [7]. However, the molecular mechanism for the induction of Rad during vascular lesion formation is unknown.

Platelet-derived growth factor (PDGF) plays an important role in normal tissue growth and the patho-physiological processes of vascular diseases like atherosclerosis and restenosis [8]. During the initiation and progression of atherosclerosis, VSMCs are activated by growth factors like PDGF or cytokines, then proliferate and migrate from the media into the intimal surface of the vessel, thus facilitating neointimal formation [8]. Egr-1 is a zinc-finger transcription factor that regulates cell proliferation and differentiation [9]. It is an immediate-early response protein that is rapidly and transiently stimulated by various growth factors including PDGF [10]. Egr-1 regulates gene transcription by the specific binding of its DNA binding domain, which consists of three zinc fingers, to the consensus GC-rich regions in the promoter of its target genes [11]. Structure analysis of Egr-1 identified a 34 amino acids inhibitory domain (R1) at the 5′ zinc finger binding region [12]. Two corepressors, NGFI-A-binding proteins 1 and 2 (NAB1 and NAB2) can markedly decrease Egr-1 transcriptional activity by binding to this domain [13], [14].

In the present study, we set out to explore how Rad is transcriptionally regulated in VSMCs. We found PDGF induced Rad expression in a dose- and time-dependent manner, which Egr-1 and its partners mediated this induction.

## Results

### PDGF induces Rad expression in RASMCs

**Figure 1:**
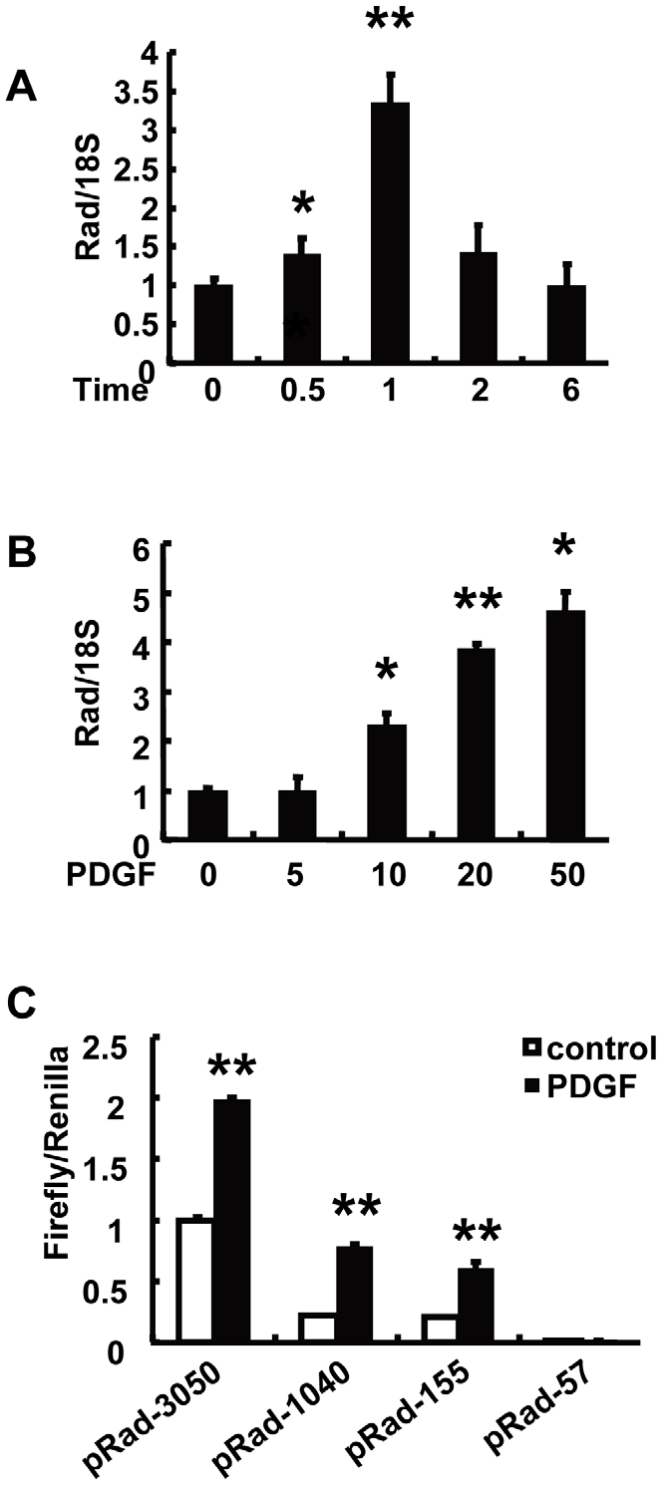
PDGF induces Rad expression in RASMCs. (A) Time course of Rad expression level in RASMCs after 20 ng/ml PDGF-BB treatment. (B) Dose-response of Rad expression after 1 hour treatment with the indicated concentrations of PDGF-BB. Rad expression was detected by real-time PCR and normalized by 18S (n = 3, * *p*<0.05, ** *p*<0.01). (C) Responses of different Rad promoter reporters to PDGF-BB stimulation in RASMCs (n = 4, ** *p*<0.01).

To determine the effects of the growth factor PDGF on Rad expression in RASMCs, we treated cultured RASMCs with PDGF (20 ng/ml) for 0, 0.5, 1, 2 and 6 hours. Quantitative real-time PCR revealed that expression of Rad increased 0.5 hours after PDGF stimulation, peaked at 1 hour, and returned to the baseline levels 6 hours later (Figure 1A). Rad was induced by PDGF in a dose-dependent manner (Figure 1B). One hour of 20 ng/ml PDGF treatment resulted in a 3.5-fold increase in Rad mRNA, compared to untreated cells (Figure 1B).

To identify the mechanisms by which PDGF activates Rad expression, we isolated Rad promoter and made reporter constructs containing different length Rad promoters. RASMCs were transfected with these constructs and then treated with PDGF. PDGF stimulated Rad promoter activity in all Rad promoter constructs except pRad-57, which also lacked basal promoter activity (Figure 1C).

### Egr-1 binding sites in the Rad promoter are required for PDGF-induced Rad promoter activation

**Figure 2:**
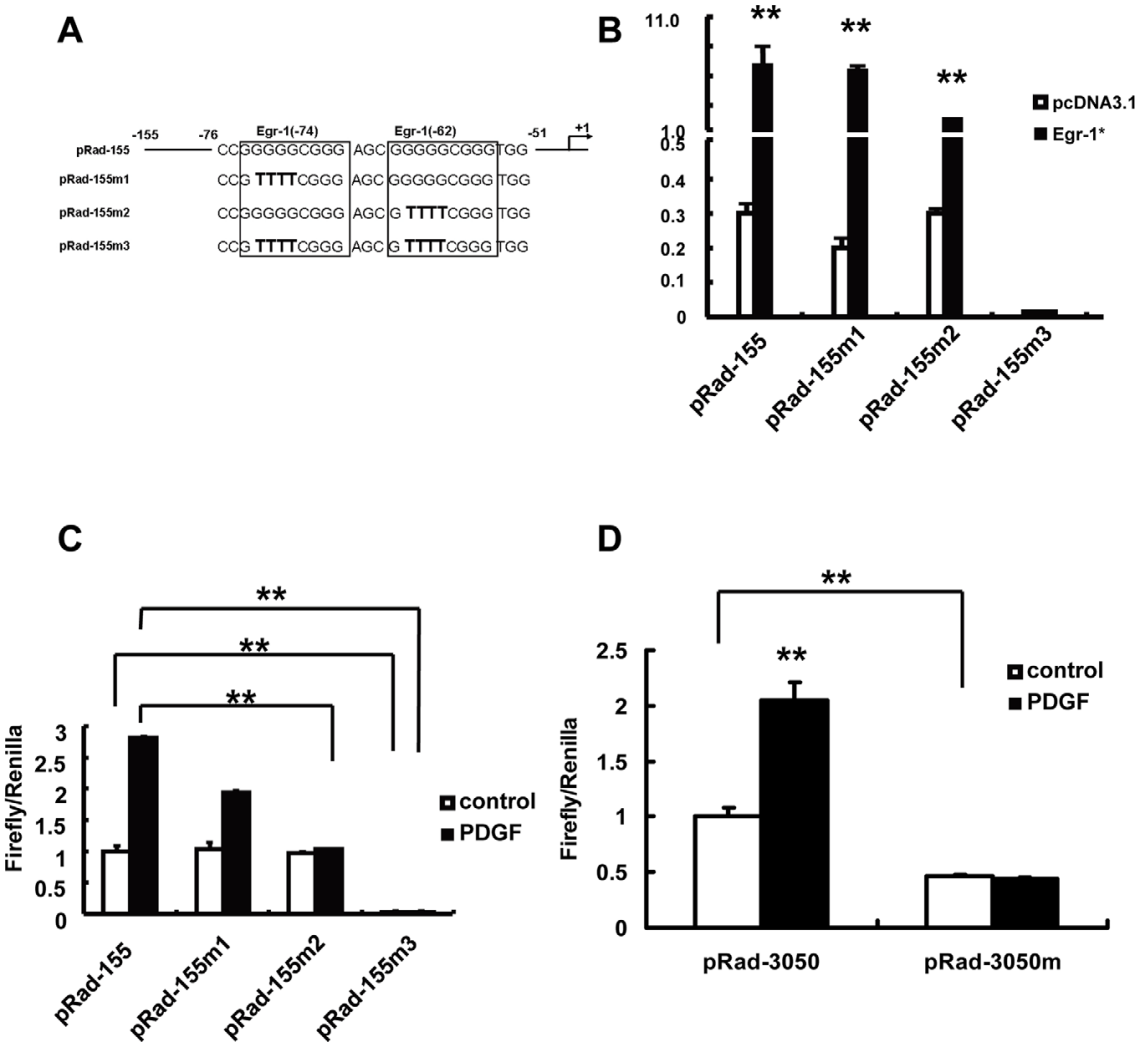
Egr-1 binding sites are essential for Rad promoter activation. (A) Nucleotide sequence between base pairs -76 and -51bp of Rad promoter. Egr-1 binding sites are boxed. The mutated bases are indicated in boldface. (B) Wild-type and mutant forms of pRad-155 were cotransfected with pcDNA3.1-Egr-1* or pcDNA3.1 into 293A cells and luciferase activity were determined. The fold induction of Lucifearse activity by Egr-1 was shown in the lower panel; (C) RASMCs were transfected with pRad-155 promoter reporter or its mutant forms then treated with PDGF and luciferase activity were determined (n = 4, **p<0.01). (D) pRad-3050 and pRad-3050m were transfected into RASMCs then treated with PDGF and luciferase activity were determined (n = 4, **p<0.01).

Computer analysis revealed two GC-rich regions that may serve as Egr-1 binding sites within the −155 to −57 bp region of the Rad promoter (Figure 2A). To investigate whether these are functional Egr-1 binding sites, we over-expressed a constitutively active Egr-1 (Egr-1*) and tested whether it could transactivate Rad promoter. Egr-1* over-expression resulted in a ∼25-fold increases of the pRad-155bp promoter activity compared with pcDNA3.1 vector transfection(Figure 2B). Then we mutated one or both of the predicted Egr-1 binding sites in the pRad-155 construct and co-transfected wild-type or mutant reporter constructs with a constitutive active Egr-1* expression construct into 293A cells. Disruption of the first Egr-1 binding site in position -74 (pRad-155m1) caused a slight decrease in the Rad promoter activity but the induction upon Egr-1* overexpression remain unchanged (Figure 2B). However, disruption of the second Egr-1 binding site in position -62 (pRad-155m2) resulted in a weaker induction of the Rad promoter activity by Egr-1* (∼7 folds) compared with the 25-fold activation in the pRad-155 construct, whereas the basal level remained unchanged. However, mutations on both Egr-1 binding sites (pRad-155m3) impaired both basal and Egr-1*-induced transactivation of the Rad promoter (Figure 2B).

To further test whether Egr-1 binding site mutation affects PDGF-induced Rad promoter activation, we transfected RASMCs with wild-type (pRad-155) or Egr-1 binding site mutant constructs and then treated the cells with PDGF. PDGF triggered a nearly 3 fold promoter activity induction on pRad-155, and the induction dropped to ∼1.8 fold in pRad-155m1, and no PDGF induction could be observed in pRad-155m2 construct meanwhile the basal activity of these constructs remained consistent. When both Egr-1 binding sites were disrupted as in pRad-155m3, the basal level of the Rad promoter activity dropped dramatically and no induction by PDGF was found (Figure 2C). These results are consistent with the above experiments performed in 293A cells overexpressing a constitutive active Egr-1*. Furthermore, mutation of both Egr-1 binding sites in the pRad-3050 reporter construct caused ∼ 54% decrease in the basal level of pRad-3050 promoter activity and PDGF-induced promoter activation was also completely abolished in pRad-3050m (Figure 2D). Taken together, the Egr-1-responsive regions located at −62 and −74 bp of Rad promoter are essential for PDGF-induced Rad promoter activation.

### Egr-1 mediates PDGF-induced Rad expression in RASMCs

**Figure 3:**
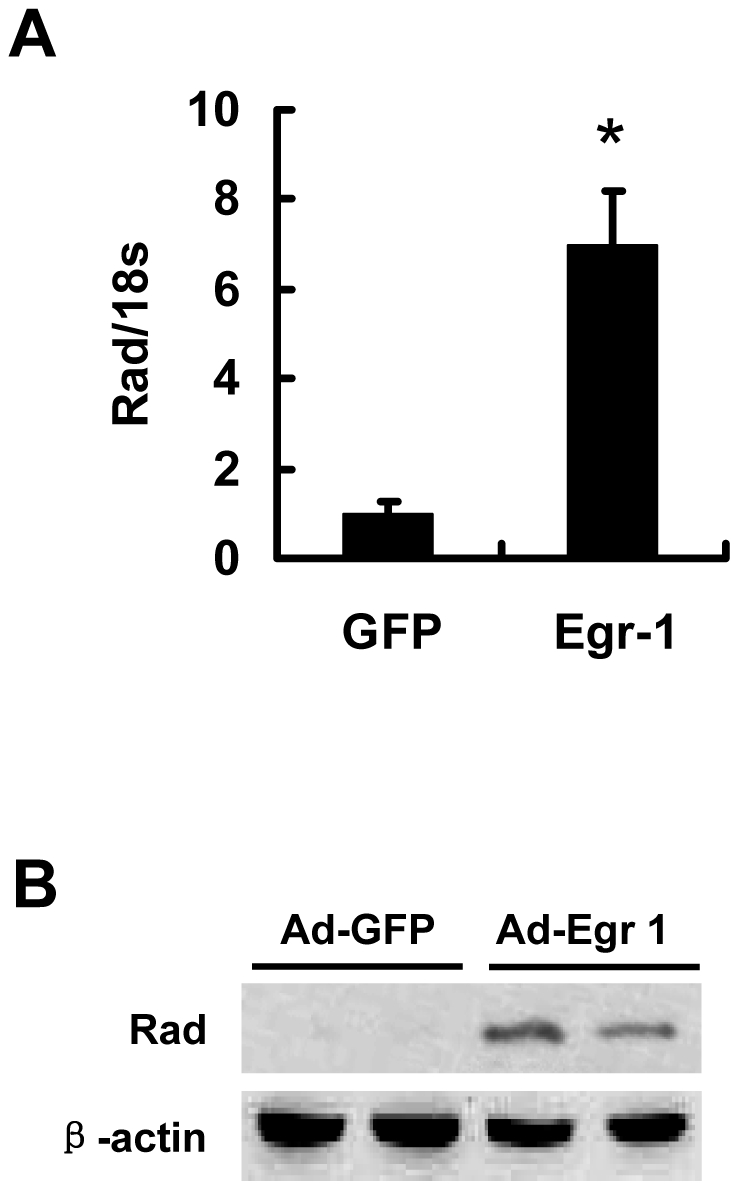
Egr-1 induces Rad expression. (A) RASMCs were infected with Ad-Egr-1or Ad-GFP for 24 hours. Rad mRNA levels were detected by real-time PCR (n = 3, **p<0.01 vs. control); (B) Rad protein levels were determined by Western blotting analysis. Expression of β-actin was detected as the control for equal loading.

**Figure 4:**
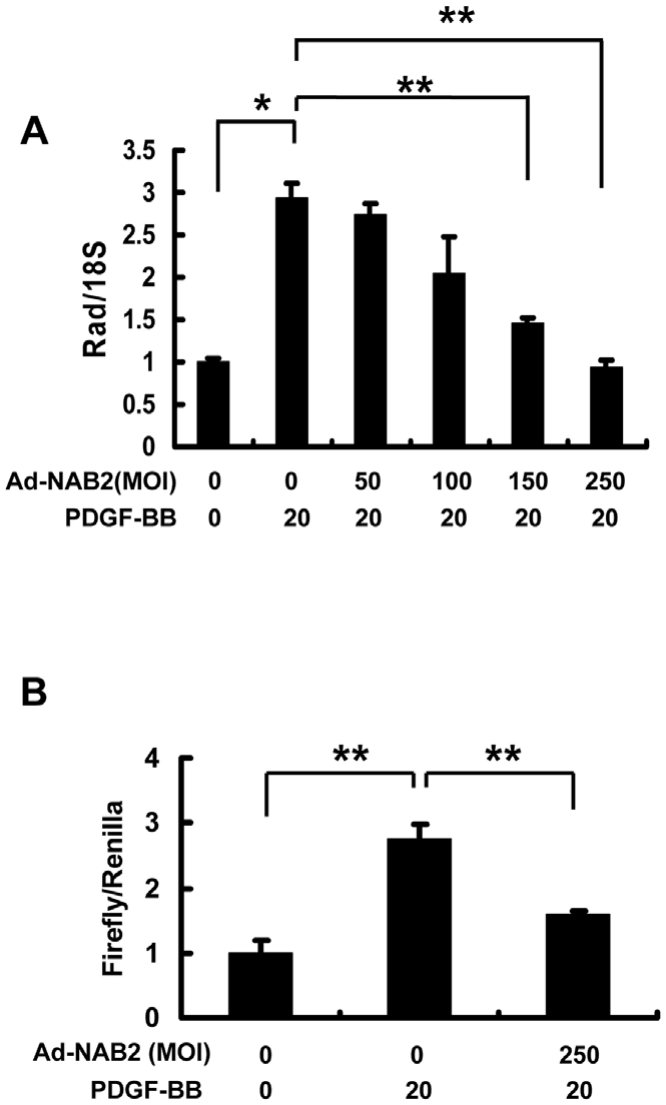
Egr-1 is an essential mediator for PDGF induced Rad expression. (A) RASMCs were infected with Ad-NAB2 at MOI of 0, 50,100,150 and 250 before treated with PDGF-BB (20 ng/ml) for 1 hour. Ad-GFP was added to keep the amount of adenovirus consistent. Rad mRNA was quantified using real-time PCR (n = 3, *p<0.05, **p<0.01); (B) RASMCs were transfected with pRad-155 and infected with Ad-NAB2 or Ad-GFP for 24 hours and then stimulated with PDGF. Luciferase activity was measured 1 hour after PDGF stimulation (n = 4, *p<0.05, ^**^ *p*<0.01).

Egr-1 was activated by various growth factors including PDGF, and we also found a remarkable induction of Egr-1 by PDGF in RASMCs (data not shown). Real-time quantitative PCR and Western blot demonstrated that Egr-1 markedly induced Rad expression at both mRNA and protein levels (Figure 3), which is consistent with Egr-1 transactivation of the Rad promoter. These results suggest that Egr-1 is a potent transcriptional activator for Rad. We hypothesized that PDGF induced Rad expression via the activation of Egr-1. NAB2 functions as a corepressor of Egr-1[14], therefore we asked whether over-expresion of NAB2 inhibits PDGF-induced Rad expression. RASMCs were infected with a recombinant adenovirus expressing NAB2 (Ad-NAB2) followed by PDGF treatment for 1 hour. Ad-GFP was used as a control in this experiment. We found that Rad expression increased in response to PDGF in Ad-GFP-infected RASMCs, and overexpression of NAB2 resulted in a dose-dependent abrogation of PDGF-induced Rad expression (Figure 4A). The induction of Rad mRNA was completely abolished when Ad-NAB2 reached a concentration of 250 MOI (multiplicity of infection). Furthermore, NAB2 inhibited Rad promoter activity elevated by PDGF-BB (Figure 4B). These results support that Egr-1 is a key mediator involved in PDGF-induced Rad expression in RASMCs.

### PDGF enhances Egr-1 binding to Rad promoter

**Figure 5:**
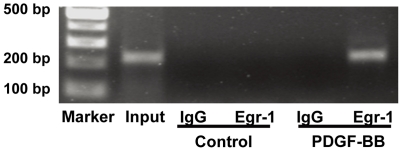
Egr-1 binds to Rad promoter after PDGF treatment. ChIP assay was performed in RASMC stimulated with or without PDGF (20 ng/ml) for 1 hour. After formaldehyde cross-linking, the protein-DNA complexes were recovered using anti-Egr-1 antibody or non-specific IgG. PCR was performed to detect the proximal Rad promoter. PCR products were detected by 2% agarose gel electrophoresis.

ChIP assay was performed to further confirm the physiological relevance and functionality of Egr-1 through its putative binding sites in the Rad promoter. RASMCs untreated or treated with PDGF for 1 hour were incubated with formaldehyde to cross-link protein and binding sites in DNA. The -213 to -2 region of the Rad promoter region was amplified by PCR. After PDGF treatment, Egr-1 was found to bind to the Rad promoter. As for DNA from untreated cells or DNA precipitated by control IgG, we did not find any PCR amplifications (Figure 5). Our data strongly support that Egr-1 bound to the proximal Rad promoter following PDGF treatment in RASMCs.

## Discussion

In the present study, we provide the first evidence that PDGF induces Rad expression in a time- and dose-dependent manner in rat aortic smooth muscle cells, and that Egr-1 is a key transcriptional factor to mediate PDGF-induced Rad expression.

Rad is a RGK-family small GTPase initially identified by subtractive cloning and found to be over-expressed in skeletal muscle of a group of patients with type II diabetes [2]. Rad possesses a structurally unique Ras-related core and COOH-and NH2-terminal extensions but lacks the CAAX-like prenylation motif at the COOH terminus possessed by Ras [15], [16]. In atherosclerotic lesions, PDGF released from inflammatory and immune cells promotes VSMCs proliferation and attracts VSMCs to migrate from media to intima [17]. In different animal models of acute arterial injury, VSMCs accumulation in lesions is inhibited by the administration of various PDGF pathway inhibitors, including neutralizing PDGF antibodies [18], PDGFR kinase inhibitors [19], and PDGFR-neutralizing antibodies [20]. Here we demonstrated that PDGF stimulated Rad expression in RASMCs in a time- and dose-dependent manner. Our previous study indicated that Rad is a critical mediator that reduces vascular lesion formation by suppressing the attachment and migration of VSMCs via inhibition of Rho/ROK activity [7]. Rad induced by PDGF may serve as a suppressor for PDGF induced smooth muscle cell migration.

We have identified that normal Rad level is critical in maintaining cardiac and blood vessel functions. Rad expression decreases in human failing hearts and Rad knockout (KO) mice are more susceptible to cardiac hypertrophy [3]. Rad inhibits myocardium L-type calcium channel activity and attenuates the β-Adrenergic Receptor (β-AR) activity [4], [5]. In blood vessel, Rad expression increases significantly after balloon injury. Unlike other small GTPase, the RGK family proteins are regulated at the transcriptional level. However, the understanding for Rad transcriptional regulation is very limited. Potential E box sequences (CANNTG), which could serve as binding sites for the HLH family transcription factors like myf5, MyoD, myogenin and MRF4, are predicted in the Rad proximal promoter region [21]. Rad expression increases significantly during skeletal muscle regeneration. Myogenic transcriptional factors like MEF2, MyoD and Myf-5 can increase the transcriptional activity of Rad promoter[22].

Until now, the molecular mechanism by which Rad is activated during vascular lesions formation is not clear. Rad promoter activity increased after PDGF treatment, suggesting that PDGF induces Rad expression at transcriptional level. Deletion and mutation analysis of Rad promoter revealed that potential Egr-1 binding sites would be critical for PDGF-induced Rad expression. It has been well documented by us and other groups that PDGF induces dramatic expression of Egr-1 [23]. Our data showed that Egr-1 activated the Rad promoter in RASMCs, and overexpression of Egr-1 resulted in a marked increase at Rad mRNA and protein levels in RASMCs. Egr-1 activity is negatively regulated by NGFI-A-binding proteins 1 and 2 (NAB 1 and NAB 2). Binding of NAB2 to Egr-1 through interaction between the NCD1 (NAB conserved domain 1, NCD1) and the R1 domain of Egr-1 represses the transcriptional activity mediated by Egr-1[14]. We found that up-regulation of Rad induced by PDGF-BB was inhibited by the co-repressor NAB2 in a dose-dependent manner. Together, these data support that Egr-1 and its interacting partner(s) are the key mediators responsible for PDGF-induced Rad expression.

Egr-1 expression is strikingly elevated in the VSMCs of atherosclerotic lesions [24] and plays critical roles in regulating VSMCs growth and intimal thickening after vascular injury [25]. Egr-1 binds preferentially to GC-rich regions of the promoters of its target genes [26]. ChIP assay indicated that after PDGF stimulation, Egr-1 binded to the proximal Rad promoter region. Serial deletions of the Rad promoter also defined a −155 to −57 bp region responsible for both basal and Egr-1-inducible promoter activity. Two GC-rich motifs were predicted in this region as potential binding sites for Egr-1. Mutation of both Egr-1 binding sites in pRad-3050 and pRad-155 resulted in dramatic reduction in Rad basal promoter activity and complete abolishment of PDGF-induced Rad promoter activation. Egr1-activated Rad promoter activity was attenuated when the putative Egr-1 binding site at position -62 or both binding sites were mutated. Consistent with this, PDGF-stimulated Rad promoter activation was abolished when the putative Egr-1 binding site in position -62 or both binding sites were mutated. These results suggest that the Egr-1 response element located at −62 bp plays the crucial role in PDGF-induced Rad promoter activity.

Here we identified Rad as another target gene of transcriptional factor Egr-1. After vascular cell injury, Egr-1 is expressed primarily in the nucleus and is capable of altering the transcription of several genes implicated in the pathogenesis of vascular disease, including PDGF, FGF-2, TNFα, tissue factor, ICAM and p53. Genes activated by Egr-1 play important roles in VSMC proliferation, neointima formation and contribute to the development of vascular diseases. Many of those genes like PDGF are activated by Egr-1 and further induce Egr-1 expression. Osteopontin and Egr-1 also positively regulate each other in VSMCs, which may play an important role in controlling inappropriate remodeling of vessel walls [27]. These positive feedback loops amplify gene transcription activated by Egr-1. On the other hand, the negative feedback loop between Egr-1 and other genes like NAB2 prevents the permanent activation of Egr-1 target genes. In response to extracellular stimuli Egr-1 induces the expression of NAB2, which in turn represses the activity of Egr-1 through binding to the R1 domain of Egr-1 [28]. Gene expression profiling by microarrray analyses revealed enhanced expression of several Egr family members in the hearts of Rad Knockout mice compared with their wild-type littermates. These raised the possibility that there is a negative feedback loop between Rad and Egr-1, i.e. Egr-1 activation induced Rad expression and Rad inhibited the further activation of Egr-1. Furthermore, the effect of Rad on other transcription factors involved in neointima formation and VSMC migration remains to be further investigated.

## Materials and Methods

### Cell Cuture

Rat aortic smooth muscle cells (RASMCs) were isolated from male Sprague-Dawley rats as described previously [23] and cultured in Dulbecco's modified Eagle's medium supplemented with 10% FBS in a 5% CO2 humidified atmosphere at 37°C. Early passages (5 to 9) of cells grown to 80-90% confluence were used for all experiments. Cells were placed in serum-free medium for 24 hours before treatment with PDGF-BB (Sigma).This study was carried out in strict accordance with the recommendations in the Guide for the Care and Use of Laboratory Animals of the National Institutes of Health. Experiments were approved by the Peking University Animal Care and Use Committee (Permit Number: IMM-XiongJW-2).

### Plasmids and adenoviral recombinants

The pcDNA3.1-Egr-1* plasmid containing constitutively active Egr-1 as well as the adenoviruses Ad-Egr-1 and Ad-NAB2 were prepared as described previously [29]. Fragments of the Rad promoter region (3050 bp, 1040 bp, 155 bp and 57 bp) were amplified by PCR and subcloned into the pGL3-basic vector to generate the pRad-3050, pRad-1040, pRad-155 and pRad-57 reporter constructs. These constructs were confirmed by DNA sequencing.

### RNA Isolation and Quantitative Real-time PCR

Total RNA was extracted from cultured RASMCs with Trizol (Invitrogen) and 1 µg was used for cDNA synthesis using a first strand cDNA synthesis kit according to the manufacturer's instructions (Invitrogen). Rad mRNA level was assessed by real-time quantitative RT-PCR as previously described [7] and 18S level was measured for normalization of variations in RNA input and cDNA synthesis.

### Western Blot Analyses

Cells were lysed with lysis buffer (Cell Signaling Technology). The lysates were resolved on 12% SDS-polyacrylamide gels, and proteins were then transferred to PVDF membranes (Bio-Rad). The membranes were blocked for 1 hour at room temperature and incubated overnight at 4°C with anti-Rad antibody (1:2000). Rabbit anti-Rad polyclonal antibody was a kind gift from Dr. Ronald Kahn [30]. Blots were then incubated with anti-rabbit secondary antibody (1∶2000) for 1 hour at room temperature and the Rad/antibody complexes were visualized by chemiluminescence (Bio-Rad) according to the manufacturer's recommendations.

### Transient Transfection and Luciferease Assays

Transfection of RASMCs or 293A cells were performed using LipofectamineTM2000 (Invitrogen) according to the manufacturer's instructions. The pRL-CMV plasmid was cotransfected as the control for transfection efficiency. Luciferase activity was measured by the Dual Luciferase Reporter System using a TD20/20 Luminometer (Turner Biosystems).

### Site-Directed Mutagenesis

Mutations were introduced in the pRad-155 or pRad-3050 plasmids using QuikChange II XL Site-Directed Mutagenesis Kit (Stratagene) according to the manufacturer's recommendations. The following oligonucleotides were used to create the desired mutations: Egr1-mut1 (for pRad-155), 5′-CCGCAGCGTGGACCG**TTTT**CGGGAGCGGGGGCGG-3′ **; Egr1-mut2 (for pRad-155), 5′-GACCGGGGGCGGGAGCG**TTTT**CGGGTGGAGGCTTAAAT-3′**; and Egr1-mut3 (for pRad-155 and pRad-3050), 5′-GGACCG**TTTT**CGGGAGCG**TTTT**CGGGTGGAGGCTTAAATA-3′ **. The mutated nucleotides are indicated in bold. Mutations were confirmed by DNA sequencing. The resulting pRad-155 mutants with one or both Egr1 binding sites mutated were designated pRad-155m1, pRad-155m2 and pRad-155m3. pRad-3050 with both Egr1 binding sites mutated was designated pRad-3050m.**

### Chromatin Immunoprecipitation (ChIP)

ChIP assay was performed using a chromatin immunoprecipitation assay kit (Upstates). Cultured RASMCs were treated with PDGF or vehicle for 1 hour. Proteins were cross-linked with DNA by 1% formaldehyde treatment for 10 minutes at 37°C. Cells were then lysed and DNA was sheared by sonication (MicrosonTM, strength 40%, pulse 10 seconds, 4 times). The sonicated DNA was diluted in ChIP dilution buffer. The diluted cell supernatant was pre-cleared with protein A agarose before immunoprecipitation with anti-Egr-1 antibody (Santa Cruz) or non-specific IgG. The immune complexes were collected by adsorption to protein A agarose, precipitated by gentle centrifugation at 3,000 rpm of 4°C for 1 min, and the supernatant containing unbound nonspecific DNA was discarded. The precipitate was washed and the immune complexes were eluted, adjusted to 200 mM NaCl and incubated at 65°C for 5 hours to reverse the crosslinks. After successive treatments with RNase A and proteinase K, the DNA was extracted with phenol-chloroform, precipitated with ethanol and resuspended in H_2_O. The immunoprecipitated DNA were analyzed by PCR with primers spanning -213 to -2 of the Rad promoter. The primer sequences were as follows: forward: 5′-TCGCTCTCTCTCTCCTTCTCACAC-3′ and reverse: 5′-ACCCTCTTCCTCGGACCTTACATC-3′. An aliquot of the sonicated DNA was used as the input. PCR products were detected by 2% agarose gel electrophoresis.

### Statistical Analysis

Each experiment was repeated for a minimum of three times. Statistics were analyzed using either ANOVA (for multiple comparisons) or Student's 2-tailed t test for comparing two means. Data are presented as Means±SD. In all cases p <0.05 was considered statistically significant.
